# Identification of U251 glioma stem cells and their heterogeneous stem-like phenotypes

**DOI:** 10.3892/ol.2013.1623

**Published:** 2013-10-11

**Authors:** SUOJUN ZHANG, RUIFAN XIE, FENG WAN, FEI YE, DONGSHENG GUO, TING LEI

**Affiliations:** Department of Neurosurgery and Chinese-German Lab of Molecular Neurooncology, Tongji Hospital, Tongji Medical College, Huazhong University of Science and Technology, Wuhan, Hebei 430030, P.R. China

**Keywords:** U251, glioma stem cells, heterogeneity, phenotype

## Abstract

Glioblastoma, the most common and lethal type of intracranial tumor, is characterized by extensive heterogeneity at the cellular and molecular levels. The discovery of glioma stem cells (GSCs) lends support to a new paradigm in tumor biology. In the present study, we aimed to clarify the validity of using U251 glioma cells as a source of GSC culture and critically evaluate the heterogeneous stem-like phenotypes of these cells when grown under various culture conditions. The findings suggested that U251 cells (U251-Adh, U251-SC-Sph and U251-SC-Adh) showed distinctive growth patterns and self-renewal capacity. The U251 glioma cell line is endowed with certain GSC phenotypes that may be moderately enriched *in vitro* when transferred into stem cell culture conditions, although this is not sustainable and reproducible *in vivo*. Notably, glioma cells are plastic in response to their environment. The reversible adaptive plasticity contributes to the GSC heterogeneity, which may lead to the heterogeneity of glioblastoma and the differing responses to current therapies. Therefore, an improved understanding of GSC heterogeneity is urgently required for designing more effective therapies against this highly malignant brain tumor.

## Introduction

There continues to be a high incidence of mortality for glioblastoma patients as a consequence of the failure of current therapies and tumor recurrence, in spite of advancements in surgery and radio- and chemotherapy (1,2). The difficulty in achieving an effective and successful treatment is mainly due to the existence of glioma stem cells (GSCs) and their heterogeneity. Besides possessing stemness characteristics similar to normal neural stem cells (NSCs), including stem cell marker expression, self-renewal and multiple differentiation potential (3–5), GSCs exhibit significant aberrations from NSCs in tumorigenicity and radio-/chemoresistance (6–9).

Glioblastoma, characterized by extensive heterogeneity at the cellular and molecular levels, shows differing growth capabilities and responses to chemo-/radiotherapy among individual cases (10,11). Similarly, the biological hallmarks of GSCs, including proliferation capacity, differentiation properties, growth phenotypes, expression of stem cell markers and regulatory mechanisms, vary considerably between individual glioblastoma cases (12–15). It is conceivable that glioblastoma heterogeneity derives, at least in part, from the existence of distinct GSC populations. This tumor heterogeneity further complicates the design of strategies for effective treatment. Therapies that would effectively eradicate gliomas would be those targeting the population of cells responsible for driving disease progression. Accordingly, the derivation and identification of bona fide GSC populations are of great concern and an improved understanding of GSC heterogeneity is required to drive the design of more effective therapies against this highly malignant brain tumor. In the present study, we aim to clarify the validity of using U251 glioma cells as a source of GSC culture and critically evaluate the heterogeneous stem-like phenotypes of these cells when grown under various culture conditions.

## Materials and methods

### Cell culture

U251 glioma cells (Cell bank of Chinese Academy of Sciences, Shanghai, China) were cultured under three growth conditions: Adherent growth in conventional 10% serum medium (U251-Adh); non-adherent sphere growth in serum-free medium (U251-SC-Sph); and adherent growth on laminin-coated flasks (Corning Inc., New York, NY, USA) in serum-free medium (U251-SC-Adh). The 10% serum medium consisted of DMEM plus 10% fetal bovine serum (FBS); while the serum-free medium consisted of DMEM/F12 supplemented with BIT (20%; Stemcell Technologies Inc., Vancouver, BC, USA), EGF (20 ng/ml; PeproTech Inc., Rocky Hill, NJ, USA) and bFGF (20 ng/ml; PeproTech Inc.) (16).

### Cell proliferation assay

Standard growth curves were plotted to compare cell proliferation rates under respective culture conditions. Cells were transferred to fresh culture flasks (25 cm^2^), according to the three culture conditions, at a density of 2×10^5^ cells/ml and passaged every three days. The growth rates of the three cell populations were analyzed by counting the cell number obtained at each passage. Growth curves were drawn according to the cell number at each passage.

### Cell cycle analysis

Cell cycle analysis was performed using a BD FACS Aria cell sorter (BD Biosciences, Franklin Lakes, NJ, USA). Cells with synchronization were maintained under the three culture conditions for 24 h and then all cells were resuspended in 70% ethanol and stored at −20ºC overnight. Prior to flow cytometry, the cells were washed with phosphate-buffered saline (PBS), centrifuged and resuspended in 0.5 μl buffer containing propidium iodide (50 μg/ml) and RNAse (100 μg/ml) for 30 min.

### Clonal formation assay

Single-cell suspensions were calibrated to 5,000 cells/ml in serum-free supplemented medium, then diluted into gradient cell titers at 1,000, 500, 200, 100, 50, 20 and 10 cells/200 μl and further transferred into the wells of a 96-well microplate. To confirm the gradient dilution results described above, more stringent clonal assays were performed by plating single cells into the 96-well plate, i.e., one viable cell per well. Clonal spheres (nonadherent, tight and spherical masses >75 μm in diameter) were counted under a microscope (Olympus CKX31, Tokyo, Japan) at the end of two weeks.

### Immunofluorescence staining

Cells were fixed with 4% paraformaldehyde and then blocked by goat serum. The primary antibodies used were rabbit polyclonal to CD133 (1/500; Abcam, Cambridge, MA, USA), mouse monoclonal to nestin (1/500; Abcam) and rabbit polyclonal to glial fibrillary acidic protein (GFAP; 1/500, Abcam). The primary antibodies were incubated for 16 h at 4ºC followed by detection with the corresponding fluorescent secondary antibodies (CD133, BA1105; nestin, BA1031 and GFAP, BA1032; Boster, Wuhan, China). Nuclei were counterstained with DAPI. Samples were subjected to evaluation under a fluorescence microscope (Eclipse TE2000S; Nikon, Tokyo, Japan).

### Relative qPCR

Total RNA was extracted from cells using an AxyPrep total RNA preparation kit (Axygen, Union City, CA, USA) according the manufacturer’s instructions. Complimentary DNA (cDNA) templates from each sample were prepared from 1 μg of total RNA primed with oligo dT primers using a First Strand cDNA Synthesis kit (Toyobo, Osaka, Japan). PCR was performed with gene-specific primers as shown in Table I, followed by 30 PCR amplification cycles (94ºC for 30 sec, annealing at correlative temperature for 30 sec and extension at 72ºC for 60 sec). Glyceraldehyde 3-phosphate dehydrogenase (GAPDH) was used as a reaction standard. Authentic bands were detected with a Gel Doc XR imaging system (Bio-Rad, Hercules, CA, USA) and determined by Quantity One software (Bio-Rad).

### Western blot analysis

The total protein concentration of the lysates was measured using the BCA Protein Assay kit (Beyotime, Haimen, China). The primary antibodies used were rabbit polyclonal to CD133 (1/500; Abcam), mouse monoclonal to nestin (1/500; Abcam) and rabbit polyclonal to GFAP (1/500; Abcam) and GAPDH (1/1,000; Boster, Fremont, CA, USA). Nitrocellulose membranes (Pierce Biotechnology, Inc., Rockford, IL, USA) were incubated with the primary antibody at 4ºC overnight, washed three times with Tris-buffered saline-Tween 20 and incubated with the alkaline phosphatase-labeled secondary antibody for 1 h at room temperature. The membranes were visualized using a DAB detection system (P0202; Beyotime). Densitometry was used to quantify the bands by Quantity One software (Bio-Rad).

### Side population (SP) assay

For the SP analysis, cells were prepared as single-cell suspensions and resuspended at 10^6^ cells/ml in prewarmed DMEM containing 2% FBS. Cells were incubated with Hoechst 33342 (Sigma, St. Louis, MO, USA) at 5 μg/ml, either alone or as a control in combination with verapamil (Sigma) at 50 μM, for 90 min at 37ºC with intermittent mixing. At the end of the incubation, cells were centrifuged at 300 × g for 5 min and resuspended in cold PBS containing 2% FBS for flow cytometry analysis. Propidium iodide (1 μg/ml) was added prior to the assay to identify dead cells. The cells were subjected to flow cytometry analysis using a BD FACS Aria cell sorter.

### Intracranial tumorigenicity

Female six- to-eight-week-old BALB/c nude mice were housed under specific pathogen-free conditions. All animal experimental protocols were approved by the Institutional Animal Care and Use Committee of Huazhong University of Science and Technology (Wuhan, China). Briefly, 1 × 10^5^ cells in 5 μl PBS were implanted stereotactically into the right basal ganglia of the nude mouse brains (coordinates, anterior-posterior +1.0 mm, medial-lateral +2.0 mm and dorsal-ventral −3.0 mm from the bregma and dura) using a 10-μl Hamilton syringe at a speed of 1 μl/min. To determine the tumorigenicity, the survival and general performance (eating, drinking and motion) of mice were monitored daily.

### Statistical analysis

Data were analyzed using SPSS 17.0 software (SPSS Inc., Chicago, IL, USA). Comparisons among the groups were performed with analysis of variance. P<0.05 was considered to indicate statistically significant differences.

## Results

### Distinctive growth patterns

**Figure 1:**
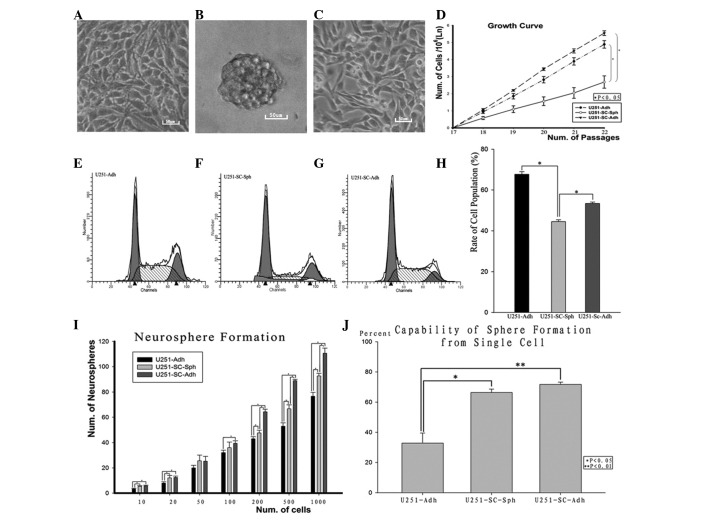
Differing growth patterns and proliferation rates of U251 glioma cells cultured under three growth conditions. (A) U251-Adh cells showed adherent growth in conventional 10% serum medium. (B) U251-SC-Sph cells showed non-adherent sphere growth in serum-free medium and (C) U251-SC-Adh cells exhibited adherent growth on laminin-coated flasks in serum-free medium. Scale bar, 50 μm. (D) Growth curves of U251 glioma cells under respective culture conditions. The horizontal axis represents the number of cell passages and the vertical axis represents the ln values, which were derived from ln_(numbers of cells/106)_. (E–G) Cell cycle analysis: Flow cytometry analysis of propidium iodide-stained U251 cells under respective culture conditions. (H) Fraction of cell population in S and G_2_-M phase. (I) Capability of tumor sphere formation from gradient cell titers at 10, 20, 50, 100, 200, 500 and 1,000 cells per 96-well plate, respectively. (J) Capability of neurosphere formation from a single cell per 96-well plate. ^*^P<0.05 and ^**^P<0.01. Adh, adherent growth; Sph, spherical growth; SC, stem cell (serum-free) medium.

GSCs were initially harvested through the neurosphere assay, growing as suspending spheres enriched with stemness characteristics. However, the adherent growth pattern has presented challenges to the classical neurosphere (17). U251 glioma cells under the three culture conditions presented distinctive growth patterns. The U251-Adh cells showed firm adherence and had elongated branches (Fig. 1A); trypsinization for passage took ~4–5 min at 37ºC. The U251-SC-Sph cells grew as floating spheres, which proliferated to 100–200 μm in diameter within 3–4 days. The cells were mechanically filtered and dissociated into single cells without trypsinization and they formed secondary spheres for serial passage (Fig. 1B). The U251-SC-Adh cells grew as an adherent monolayer in the laminin-coated flask and exhibited shorter cellular branches (Fig. 1C) and required only 2–3 min at 37ºC for trypsinization.

### Differential proliferation rates and self-renewal capacity

The proliferation capacity of GSCs varies considerably between individual GBM cases. With regard to the U251 glioma cell line, the proliferation rate varied between the cells as demonstrated by growth curve plots (Fig. 1D). U251-Adh and U251-SC-Adh cells showed a higher rate of proliferation compared with U251-SC-Sph cells (P<0.05). Correspondingly, cell cycle analysis revealed that the two types of adherent cells exhibited an increased population of cells in S and G_2_-M phase compared with that of U251-SC-Sph cells (Fig. 1E–H).

Self-renewal capacity is one of the most important stem cell phenotypes. As manifested by the limiting dilution cloning assay and further supported by the more stringent single-cell sphere formation assay, the U251-SC-Sph and U251-SC-Adh cells grown in serum-free medium possessed increased self-renewal potentiality compared with that of the U251-Adh cells grown in 10% serum medium (Fig. 1I–J).

### Discriminatory expression of cell markers

**Figure 2:**
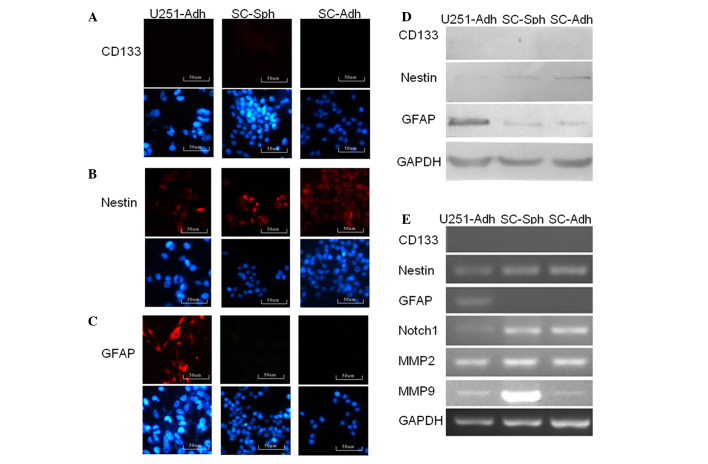
Discriminatory cell marker expression of U251 cells under three culture conditions. (A–C) Immunofluorescence analysis of CD133, nestin and GFAP expression. Although a lack of immunoreactivity was observed for CD133, a higher expression of nestin was found in U251-SC-Sph and U251-SC-Adh cells. While a higer expression of GFAP was detected only in U251-Adh. The lower rows showed Nuclei counterstained with DAPI. (D) Western blot analysis of CD133, nestin and GFAP expression. (E) RT-PCR analysis of CD133, nestin, GFAP, Notch1 MMP2 and MMP9 expression. Adh, adherent growth; Sph, spherical growth; SC, stem cell (serum-free) medium; GFAP, glial fibrillary acidic protein; MMP, matrix metalloproteinase; GAPDH, glyceraldehyde 3-phosphate dehydrogenase.

To determine the immunophenotype of the three types of cells, their immunoreactivity for markers of stem cells and differentiated cells was detected. For the analysis of CD133, nestin and GFAP expression, immunofluorescence, western blotting and relative qPCR were used. Although all three types of cells exhibited a lack of immunoreactivity for CD133, U251-SC-Sph and U251-SC-Adh cells showed higher expression of nestin at the protein and RNA levels compared with that of U251-Adh cells. GFAP is a marker of differentiated neural cell type for astrocytes. Notably, U251 glioma cells under the three culture conditions exhibited distinguishing immunoreactivity for GFAP. Compared with U251-SC-Sph and U251-SC-Adh cells, U251-Adh cells showed statistically significant immunoreactivity for GFAP. There was no statistical difference in the immunoreactivity for GFAP between U251-SC-Sph and U251-SC-Adh cells (Fig. 2).

Notch1 activity was detected in the three types of cells. Notably, the RT-PCR analysis demonstrated that Notch1 expression at the mRNA level was noticeably higher in U251-SC-Sph and U251-SC-Adh cells, compared with that in U251-Adh cells (Fig. 2E). This suggested that Notch1 may have important roles in maintaining the stemness of GSCs.

### Matrix metalloproteinases (MMPs) degrade the extracellular matrix and create a more permissive environment for cell invasion

We aimed to investigate the presence of heterogeneity in MMP-2/9 mRNA expression by means of RT-PCR analysis. Elevated levels of MMP-2/9 were detected in U251-SC-Sph, as well as U251-SC-Adh cells compared with U251-Adh cells, indicating a role in malignant progression (Fig. 2E). It followed that culture under serum-free and supplemented media gave U251 glioma cells higher migration potential.

### Distinct SP ratios

**Figure 3:**
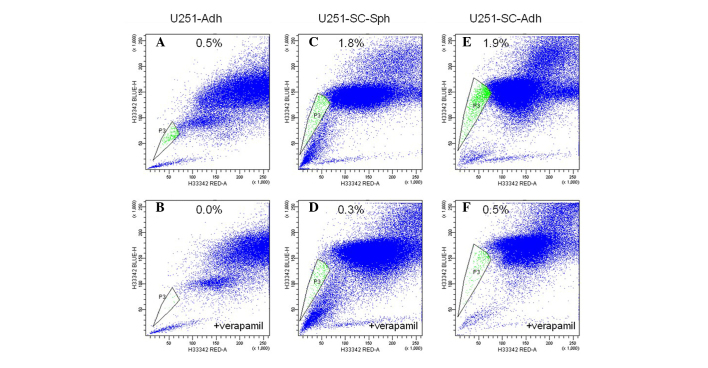
SP analysis. (A, C and E) Flow cytometry analysis of SP cells under respective culture conditions. (B, D and F) The cells were treated with 50 μM verapamil during the labeling procedure. SP, side population; Adh, adherent growth; Sph, spherical growth; SC, stem cell (serum-free) medium.

**Figure 4:**
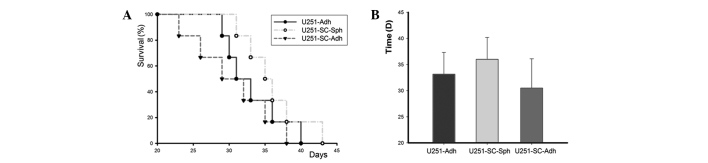
Differences in tumorigenicity. (A) Kaplan-Meier blot showing survival of nude mice and (B) mean survival time of mice after being injected with U251 cells under respective culture conditions. Adh, adherent growth; Sph, spherical growth; SC, stem cell (serum-free) medium.

To determine whether the U251 cells grown under the three culture conditions had different SP ratios, their capacity to extrude Hoechst 33342 dye was detected by flow cytometry. As shown in Fig. 4, SP cells existed in all three populations, but only a small proportion of cells possessed the ability to extrude Hoechst 33342 dye. Distinct SP ratios were observed among these three types of cells. U251-SC-Sph and U251-SC-Adh cells cultured in serum-free supplemented media showed enhanced SP ratios, compared with U251-Adh cells in serum-containing medium (Fig. 3). However, there was no statistically significant difference of SP ratios between U251-SC-Sph and U251-SC-Adh cells. The above results suggest that SP cells are enriched under serum-free supplemented medium.

### Differences in tumorigenicity

To determine the tumorigenicity of U251 glioma cells under various culture conditions, three types of cells were implanted stereotactically into the right basal ganglia of nude mouse brains. Tumor-burdened mice that developed weight loss >10% or neurological signs were recorded. U251-SC-Adh cells were more oncogenic with shortened survival (Fig. 4A), although statistically significant differences in median survival time were not confirmed (Fig. 4B).

## Discussion

The majority of current glioma research is focused on cellular and molecular analysis of the bulk tumor mass. However, the GSC hypothesis posits that a subpopulation of cells within gliomas has true clonogenic and tumorigenic potential (18). The identification of GSCs provides a powerful tool to investigate the tumorigenic process and develop therapies targeted against GSCs. Significantly, a more controversial correlation with GSCs is that cells in different culture conditions may display heterogeneous stem-like properties (17,19,20). Considering these possibilities, the present study compared the stem-like properties of U251 glioma cells under three culture conditions, to clarify the validity of using established cell lines as a source of GSC culture and to critically evaluate and quantitatively compare the stem-like phenotypes of the cells grown under various culture conditions.

The present study showed that there was a small subpopulation of cells with certain GSC phenotypes in the U251 glioma cell line, which were moderately enriched *in vitro* when transferred into stem cell culture conditions. These three types of GSC subpopulation possessed heterogeneous stem-like phenotypes, which were dependent on culture conditions. Regardless of the distinct growth patterns between the three groups of cells, stem-like characteristics existed in all three types, although at varying levels.

Self-renewal capability is the essential characteristic of tumor stem cells. The *in vitro* neurosphere formation capability has been applied to identify the self-renewal of GSCs and is considered to be a significant and independent predictor of clinical outcome of glioma patients (21). Notably, the present study demonstrated that the dominant determinant of cell self-renewal capability is the stem cell medium, i.e., serum-free medium supplemented with mitogens, rather than adherent or sphere growth patterns. It is noteworthy that the increased self-renewal capability was more remarkable between U251-SC-Adh and U251-Adh cells, compared with that between U251-SC-Sph and U251-Adh cells, indicating that adherent growth in stem cell medium provided the most favorable conditions for cell self-renewal, which is in accordance with the study by Pollard *et al*(17). It remains to be determined whether the distinct growth patterns and self-renewal capabilities *in vitro* implicate the various GSC subpopulations *in vivo* or merely an *in vitro* reversible adaptive plasticity under microenvironment manipulation.

CD133 defines a broad population of somatic stem and progenitor cells, including NSCs and GSCs (18,22,23). CD133 is considered the most important GSC marker identified to date (24–27), although there are emerging paradoxes, such as CD133^−^ GSCs may exist (28,29). In the present study, CD133 immunoreactivity was completely negative in all three cell types and the results were further confirmed by western blotting and PCR. However, nestin immunoreactivity was positive among all the cell types and there was no differential expression between different types. GFAP expression was significantly downregulated in cells cultured in the stem cell medium regardless of whether they grew adherently or as spheres.

The ability to exclude the Hoechst 33342 fluorescent dye from the intracellular compartment, originally developed by Goodell *et al*(30), is identified as a valid marker-independent method of identifying GSCs (31–33). In the present study, a small fraction of SP cells was detected in the conventionally cultured U251 cells and this SP fraction increased in cells cultured in stem cell medium. However, unresolved issues concerning the potential toxicity of Hoechst 33342 to non-SP cells limits the further application of this functional assay to the identification of GSC subpopulations, and there are also other intrinsic limitations of the assay itself (19).

Several cellular signaling pathways that regulate the self-renewal of normal and tumor stem cells have been identified and may serve as targets against GSCs. The Notch signaling pathway is important in the regulation of GSC proliferation and cell differentiation (34–36). High expression levels of Notch signaling have been detected in CD133^+^ cell fractions and SP, while Notch blockade reduced the CD133^+^ cell and nestin^+^ cell fraction (37). In the present study, Notch1 expression at the mRNA level was notably enhanced in the U251-SC-Sph and U251-SC-Adh cells, compared with that in the U251-Adh cells, which was in accordance with the differences in their self-renewal capacities. These findings were also in line with the MMP expression results in all three types of cells. Increased expression of MMPs was observed in the U251-SC-Sph and U251-SC-Adh cells, which may contribute to the enhanced invasive and migratory potential of GSCs.

Tumorigenic potential is the final and critical criteria for the identification of the GSCs. Notably, *in vivo* assays of the three types of cells revealed no significant differences in their tumorigenicity, regardless of the distinct *in vitro* stem-like phenotypes. Thus, it is deducible that the *in vitro* stemness alterations observed in the cells cultured in serum-free medium may only be reflective of epigenetic phenomena resulting from the artificial manipulation of *in vitro* growth conditions, rather than being sustainable and reproducible *in vivo*. It is possible that tumor cells possess the ability to adapt to the *in vitro* environment. This adaptation is most frequently described as a process of selection of the fittest tumor cell clones generated by mutations as a consequence of genetic instability. There is also increasing evidence for the critical roles of the GSC microenvironment (also termed niche) in maintaining and regulating the GSC phenotypes (15,38,39). Accordingly, the GSC microenvironment contributes to the GSC heterogeneity, which increases the difficulty of effectively targeting the neoplastic population responsible for tumorigenesis.

In conclusion, the present study critically evaluated and compared the stemness phenotypes of U251 cells grown under three culture conditions. The findings suggest that the U251 glioma cell line is endowed with certain GSC phenotypes that are moderately enriched *in vitro* when transferred in to stem cell culture conditions, which is, however, not sustainable and reproducible *in vivo*. Notably, glioma cells are plastic in response to their environment. This reversible adaptive plasticity may contribute to the GSC heterogeneity and their differing responses to current therapies *in vitro*.

## Figures and Tables

**Table I:** Primers used in this study.

Primer	Sense primer	Antisense primer	AT (ºC)
CD133	CCTCTGGTGGGGTATTTCTT	AGGTGCTGTTCATGTTCTCC	55
Nestin	AGGATGTGGAGGTAGTGAGA	TGGAGATCTCAGTGGCTCTT	57
GFAP	GCAGAGATGATGGAGCTCAATGACC	GTTTCATCCTGGAGCTTCTGCCTCA	60
Notch1	CCGCAAGCCCAGCAGCAAA	GGACCCGCCCACAGTGAAAT	52
MMP2	GGCCCTGTCACTCCTGAGAT	GGCATCCAGGTTATCGGGGA	55
MMP9	TGCAACGTGAACATCTTCGACGC	TCCTCAAAGACCGAGTCCAGCT	55
GAPDH	CGAGAAATATGACAACTCCCTCA	GCCTGCTTCACCACCTTCTT	59

GFAP, glial fibrillary acidic protein; MMP, matrix metalloproteinase; GAPDH, glyceraldehyde 3-phosphate dehydrogenase; AT, annealing temperature.
